# Aging and Age-Related Epigenetic Drift in the Pathogenesis of Leukemia and Lymphomas: New Therapeutic Targets

**DOI:** 10.3390/cells12192392

**Published:** 2023-09-30

**Authors:** Alessandro Allegra, Santino Caserta, Giuseppe Mirabile, Sebastiano Gangemi

**Affiliations:** 1 Division of Hematology, Department of Human Pathology in Adulthood and Childhood “Gaetano Barresi”, University of Messina, Via Consolare Valeria, 98125 Messina, Italy; santino.caserta@polime.it (S.C.); giuseppe.mirabile@polime.it (G.M.); 2 Allergy and Clinical Immunology Unit, Department of Clinical and Experimental Medicine, University of Messina, Via Consolare Valeria, 98125 Messina, Italy; gangemis@unime.it

**Keywords:** aging, epigenetics, epigenetics clock, epigenetics drift, hematopoiesis, leukemia, lymphoma

## Abstract

One of the traits of cancer cells is abnormal DNA methylation patterns. The idea that age-related epigenetic changes may partially explain the increased risk of cancer in the elderly is based on the observation that aging is also accompanied by comparable changes in epigenetic patterns. Lineage bias and decreased stem cell function are signs of hematopoietic stem cell compartment aging. Additionally, aging in the hematopoietic system and the stem cell niche have a role in hematopoietic stem cell phenotypes linked with age, such as leukemia and lymphoma. Understanding these changes will open up promising pathways for therapies against age-related disorders because epigenetic mechanisms are reversible. Additionally, the development of high-throughput epigenome mapping technologies will make it possible to identify the “epigenomic identity card” of every hematological disease as well as every patient, opening up the possibility of finding novel molecular biomarkers that can be used for diagnosis, prediction, and prognosis.

## 1. Introduction

### 1.1. Epigenetics of Cancer in Relation to Aging 

The natural biological process of aging is characterized by a steady loss in tissue and organ function. Another definition of it is the inescapable, time-dependent deterioration in the structural integrity of organ physiology. As consequences of the activity of environmental risk factors like smoking, chemicals, and stress, progressive modification of substances at the molecular level causes a rise in the risk of many chronic diseases and disabilities [1]. 

One of the main risk factors for the onset and advancement of cancer is aging, along with accompanying comorbidities like obesity, smoking, drinking alcohol, and telomere shortening. As a result, there is a close connection between aging and cancer. The former is thought to be one of the major causes of the latter, and cancer is regarded as an age-related disease because its incidence rate increases with increasing age: in the United States, people 55 and older account for 80% of all cancer cases [2]. Additionally, individuals with older age typically have inferior performance status and a worse prognosis for certain cancers. 

**Figure 1:**
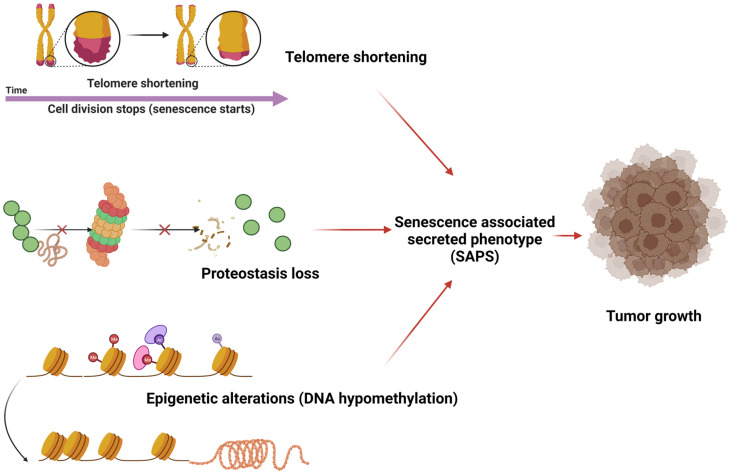
Aging-related changes, including telomere shortening, proteostasis loss, and DNA hypomethylation, promote the senescence-associated secreted phenotype (SASP) and, in turn, the proliferation of neoplastic cells.

In some instances, it is also discovered that aging and cancer have similar mechanisms. Genomic instability, telomere attrition, proteostasis loss, diminished nutrition sensing, altered metabolism, and epigenetic alterations are a few examples of these pathways [3,4]. Because cancer and aging are closely related, it is apparent that epigenetic factors influence both diseases’ entire development and course. Numerous external factors, such as the environment, pollution, lifestyle choices, and the type and amount of food consumed, have an impact on different epigenetic pathways. They are also thought to be essential for gene expression. Recent genome-wide epigenetic studies have uncovered specific epigenomic characteristics that are shared by cancer and aging [5,6]. There is mounting evidence that senescent cells may contribute to oncogenesis, given that the risk of developing cancer increases with aging where senescent cells are accumulating [7]. Senescence does represent a potent tumor suppressor mechanism imputable to the permanent cell cycle arrest. Furthermore, it has been shown that senescent human fibroblasts can induce tumors in animals and favor the stimulation of premalignant and malignant epithelial cells to grow in culture over normal epithelial cells [8]. In addition, it is possible that the senescence-associated secretory phenotype (SAPS), a phenotype linked to senescent cells, promotes tumor cell development, invasion, and metastasis, and tumor vascularization by secreting inflammatory cytokines, immune modulators, and growth factors [9]. Numerous studies have revealed relatively substantial similarities between cancer cells and senescent cells in terms of DNA methylation. Both DNA methylation and chromatin modifications exhibit this overlap. In actuality, genetic instability and the repression of tumor suppressor genes, which are typically present in cancer cells, are mechanistically linked with the characteristic scenario of decreasing genome-wide DNA methylation and the presence of site-specific DNA hypermethylation identified in senescent cells, respectively [10] (Figure 1).

It is well known that aging causes changes in genomic DNA methylation [11]. While certain alterations are crucial to development, others happen randomly and seem to have no biological purpose [12]. These molecular changes, often referred to as epigenetic drift, are being studied since it has been suggested that they may be responsible for several age-related illnesses [13]. A collection of gene promoters in blood that get hypermethylated with aging has been discovered in several recent investigations employing 1.5 K and 27 K Illumina methylation arrays [14]. Interesting studies have also revealed that many of these DNA sequences are hypermethylated in cancer and are enriched in repressive histone marks like H3K9me3 and H3K27me3 [15,16].

However, the recent invention of epigenetic clocks [17] has given us a new perspective with which to investigate aging-associated epigenetic dysregulation and its link with carcinogenesis, in addition to the canonical hyper- and hypomethylation indicators of aging and cancer.

Numerous indicators, risk factors for diseases, and health outcomes have been linked to epigenetic clocks, each of which captures a different facet of the aging process. These clocks can be separated into intrinsic and extrinsic groups. Extrinsic aging is affected by cell type proportion and environmental influences, but intrinsic aging is independent of cell type and partially driven by cellular division [18].

Epigenetic clocks are mathematical methods that use observed DNA methylation levels at various CpG sites in a subject’s genome to forecast their chronological age with high accuracy [17]. These algorithms capture a combination of chronological and biological age, the latter being a measure of an individual’s “healthiness” in terms of their risk of developing age-associated adverse outcomes [19]. There is now convincing evidence of the association between changes in the predictions made by epigenetic clocks and lifestyle factors, disease—including cancer—or outright mortality [20,21].

For instance, supercentenarian or long-lived subjects exhibit younger epigenetic ages in addition to reduced incidence or delayed start of illnesses [22,23].

Additionally, there is emerging evidence that human healthcare interventions based on pharmaceutical or lifestyle choices might rejuvenate the epigenetic clock [24,25,26].

The results of a recent study that examined the genome-wide DNA methylation status in newborns, middle-aged people, and centenarians supported those from the methylation arrays and demonstrated that overall hypomethylation, which primarily affects repetitive DNA sequences, is linked to aging [15]. Bork et al. used 27 K methylation arrays to examine the DNAm status of mesenchymal stem cells (MSCs) obtained from young and old donors, and discovered similar DNA methylation changes over time during prolonged in vitro culture and in vivo aging [27]. However, some studies have examined DNAm during aging in human adult stem cells and, using the same methylation arrays, found hypomethylation of differentiation-associated genes as well as de novo methylation events suggesting epigenetic alterations in older hematopoietic progenitor cells [28]. The functional reduction of hematopoietic stem cells (HSCs) as people age has been linked to several genome-wide changes in DNA methylation, according to recent studies in mice [29,30].

A different study found 18,735 hypermethylated and 45,407 hypomethylated CpG sites connected to aging when DNA methylation profiling of MSCs taken from people aged 2 to 92 years was performed [31]. Hypermethylated sequences were enriched in chromatin repressive markers, just like in differentiated cells. The active chromatin mark H3K4me1 was significantly overrepresented at hypomethylated CpG sites in stem and differentiated cells, demonstrating that this is a cell type-independent chromatin signature of DNA hypomethylation throughout aging. In MSCs and differentiated cells, analysis of scedasticity revealed that interindividual variability of DNA methylation increased with aging, opening up a new route for the detection of DNA alterations across time. Genetically identical individuals’ DNA methylation profiles revealed that nongenetic as well as genetic factors affected DNA methylation propensity and scedasticity. These findings show that the DNA sequence, cell type, and chromatin environment all play important roles in the dynamics of DNA methylation during aging.

### 1.2. Mechanisms of Aging- and Cancer-Related Epigenetic Drift

As previously mentioned, the term “epigenetic drift” refers to all alterations that have a broad impact on the epigenome [32]. Age-related changes in the epigenome and transcriptome landscape were demonstrated by Benayoun et al. to induce inflammatory responses, primarily interferon-related ones, in a variety of tissues [33]. Peripheral blood mononuclear cells from individuals of various ages have been used to study epigenetic drift in humans [34,35].

In order to determine the degree and interindividual heterogeneity in age-related changes in DNA methylation at particular CpG islands, researchers analyzed 377 volunteers who were 85 years old [36].

Even at well-known tumor suppressor genes like TWIST2, they discovered broad and highly variable methylation of promoter-associated CpG islands with levels ranging from 4% to 35%. Furthermore, this older population’s interindividual variations in methylation exhibit numerous characteristics of the altered methylation patterns found in cancer cells. Both methylation associated with aging and methylation associated with cancer can occur at identical sets of genes, lead to densely methylated, and probably transcriptionally repressed, alleles, and demonstrate coordinated methylation across many loci. Additionally, during a 3-year follow-up period, elevated methylation levels were linked to a subsequent leukemia or lymphoma diagnosis [36]. These findings imply that the increased cancer risk associated with aging may be a result of the accumulation of aging-related alterations in promoter-associated CpG islands.

Expectedly, aging is a key risk factor for a number of hematologic syndromes and cancers, including acute myeloid leukemia (AML) and myelodysplastic syndromes (MDS) [37]. Additionally, hemopoiesis and the ability of hemopoietic stem cells (HSCs) to regenerate are negatively impacted by age.

### 1.3. Aging and Hemopoiesis

The question of how HSCs can withstand the effects of aging has been the subject of numerous investigations. HSCs are a rare cell population that, for the most part, remain dormant and undergo very few divisions over the course of an organism’s existence, making it extremely difficult to study how they operate in living things [38,39]. Changes in the niche—which are extrinsic—and the HSC—which are solely dependent on the stem cell itself—occur during aging.

Both aging-related epigenetic processes and aging per se have an impact on hematopoiesis. In fact, compared to youthful HSCs, distinct alterations in the epigenome and chromatin organization take place in aged HSCs. These alterations involve chromatin rearrangement [40,41], particular histone posttranslational modifications, and DNA methylation [30].

The function of hematopoietic stem cells gradually declines as an individual ages normally, depending on both cell intrinsic and external stimuli [42,43]. Alterations in cell polarity, genomic integrity, and the epigenetic landscape that come with aging are the main causes of altered HSC function, but the aged BM microenvironment also contributes in significant ways [44,45].

Genome-wide expression analysis of young and old mouse HSCs has demonstrated that aging-related transcriptional changes in HSCs affect myeloid and lymphoid differentiation [46,47].

Most somatic cells as well as primordial cells have global hypomethylation as they age [48,49]. Nevertheless, the overall methylation difference between elderly and young HSCs varies depending on the study. Age-related hypermethylation was detected in research using reduced-representation bisulfite sequencing. In contrast, MeDIP-seq data from Taiwo et al. revealed a small but significant (5%) worldwide decrease in DNA methylation. According to Taiwo et al., the variation could be explained by the disparity in coverage between the two techniques [29].

HSCs’ adult-life quiescence is one potential explanation for the restriction to a more widespread and conspicuous hypomethylation on HSCs throughout aging. Global hypomethylation happens when young or aged mouse HSCs are transplanted, causing them to experience an induced proliferative stress. This finding is consistent with studies on the aging of human somatic cells and tissues. In spite of this, mice can still identify the hypermethylation of PRC2 targets, indicating that age and tiredness cause different changes to the DNA methylation landscape.

One of the most precise investigations into the epigenomic changes brought on by aging in murine HSCs was carried out by Goodell’s team [41]. ChIP-seq was used to profile some of the key regulatory histone marks in HSCs from 4- and 24-month-old mice. Even though the dataset only showed minor variations, some intriguing special features were reported. For instance, there was only a 6.3% increase in peak deposition of H3K4me3 in old versus young HSCs but the majority of the peaks were generally wider. Peak levels for H3K27me3 remained stable but the length of coverage increased by 29% with HSC aging.

In addition, the signal’s strength rose by 50%. With regard to H3K36me3, the peaks shifted from the transcription start site to the transcription termination site, and H3K36me3 and H3K27me3 behaved antagonistically. The three histone marks examined in the study showed a high positive connection with transcriptome alterations [41]. Regarding other histone markers, it has been shown that the levels and location of H4K16ac alter with aging in murine HSCs. By using a single cell, three-dimensional immunofluorescence analysis, young HSCs demonstrate a notable distribution of H4K16ac in one pole of the nucleus. The term “epigenetic polarity” or “epipolarity” is used to describe this quality. H4K16ac levels in older HSCs are lower; they also exhibit apolarity and localize uniformly across the nucleus [50,51,52]. In murine HSCs and ST-HSC, the polarity and amounts of a number of additional histone marks, including H3K4me1, H3K4me3, H4K8ac, H3K27ac, and H4K5ac, did not alter with age. It is interesting to note that global alterations in gene expression did not primarily correlate with the loss of epipolarity and the decrease in global H4K16ac deposition following aging in HSCs. Inhibiting Cdc42 activity and LaminA/C expression was shown to target H4K16ac, which was linked to the modification of higher-order chromatin and protein interactions between chromatin and non-histone proteins [53].

A few hematopoietic lineages that were themselves descended from HSCs have been documented to contribute to the regulation of HSCs in addition to the BM cell types mentioned above. Megakaryocytes have been demonstrated to cause HSCs to go into quiescence [54], while regulatory T cells and macrophages have been shown to alter HSC survival [55,56].

A natural temporal decline in HSC activity may be influenced by age-related changes in the composition, proliferative ability, spatial arrangement, expression of adhesion molecules, and secretome of niche cells [57,58].

## 2. Clonal Hematopoiesis

Following the acquisition of somatic driving mutations, clonal hematopoiesis (CH), consisting in the growth of hematopoietic stem and progenitor cell (HSPC) clones and their progeny, takes place. Clonal hematopoiesis of indeterminate potential (CHIP) patients do not have abnormal blood cell counts or any other hematologic disease symptoms but do have somatic mutations in hematological malignancy-associated driver genes, historically at or above a variant allele frequency of 2% [59]. However, CHIP is linked to a slightly elevated risk of hematological malignancy as well as a higher chance of developing cardiovascular and pulmonary disease.

According to estimates, human HSPCs experience an average of 1 exonic mutation every decade of life, so that, by the age of 50, each HSPC would have accumulated an average of five coding mutations [60]. Inferences about lineage linkages and population dynamics in human hematopoiesis have been made as a result of two other studies’ observations of the steady acquisition of mutations in HSPCs with aging [61,62]. Although the majority of these mutations are most likely to be neutral, a mutation that affects an HSPC clone’s ability to survive selection will be overrepresented and may result in CH.

Early evidence for age-related CH came from studies of X-chromosome inactivation across time [63,64,65], which established the link between CH and aging. A small cohort of older people was also shown to have somatic TET2 mutations in the absence of hematological illness [64]. Large-cohort studies were employed by several groups to discover the accumulation of particular mutations in the blood of healthy aged individuals at surprisingly high prevalence and a strong association with unfavorable outcomes, as discussed below. This was made possible by the development of more widely available high-throughput sequencing [65]. TP53, PPM1D, SRSF2, SF3B1, signaling (JAK2), and other epigenetic regulators (BCORL1) were also found to be mutated, in addition to the epigenetic regulators DNMT3A, TET2, and ASXL1, which are the most frequently affected. Contrary to popular belief, aged mouse blood does not frequently contain mutations in TET2 and DMNT3A, despite the fact that murine models with these mutations mimic many CH symptoms [66,67,68,69].

The impact of aging and epigenetic aging on the beginning and development of hematologic disorders will be examined in the sections that follow.

## 3. Leukemia

### 3.1. Acute Myeloid Leukemia

According to the National Institutes of Health and Surveillance, Epidemiology and End Results database, the typical age at diagnosis for acute myeloid leukemia is 68 years old, like many other cancers. Even though leukemia research has been studied for more than 50 years, senior AML patients’ long-term survival rates are still shockingly poor [70].

A large percentage of AML patients have mutations in epigenetic modifiers [71]. DNA methylation, DNA hydroxymethylation, histone acetylation, and histone lysine methylation are the epigenetic alterations that are most frequently described in the context of AML [72].

To define aging-associated changes and explain why older people are more likely to develop cancer, numerous studies have been conducted [73,74,75,76]. For various tissues, experiments have shown that the frequency of mutations is two to three times higher in aged cells than in young or young adult cells [77,78]. Quiescence of these cells, which results in DNA repair attenuation, has been hypothesized to be one of the causes of the accumulation of mutations in HSCs [79]. Furthermore, it was demonstrated that HSCs could repair DNA damage regardless of age when the cell cycle was stimulated [79]. According to Moehrle et al. [80], who demonstrated that both young and old HSCs prefer to exit their quiescent condition upon DNA damage in vivo, this concept has been consistently validated. It is interesting to note that the same study found that, in comparison to youthful HSCs, the ability of elderly HSCs to repair DNA damage was unaffected [80].

The ability to accurately estimate chronological age using DNA methylation patterns has been demonstrated by large-scale methylome research [81,82]. Interesting evidence reveals that DNA methylation age is an intrinsic attribute of the cell and is unaffected by the environment because donor HSCs transplanted into recipient patients of varied ages have been shown to maintain their chronological DNA methylation age [83].

Furthermore, numerous investigations found that aged hematopoietic cells had altered expression of epigenetic regulators as well as a variety of epigenetic modifications [84,85].

For instance, a thorough investigation of the transcriptome, DNA methylome, and histone modifications in young and old mouse HSCs revealed worldwide epigenetic alterations linked to stem cell aging. The authors showed changed placement of several regulatory histone marks such H3K4me3, H3K27me3, and H3K36me3 as well as decreased production of DNA methyltransferases, the main epigenetic regulators, in old cells.

Furthermore, Adelman et al.’s [86] examination of the epigenetic landscape of an HSC-enriched population from young and elderly healthy donors indicated a drop in H3K4me1, H3K27ac, and H3K4me3 with aging, as well as altered DNA methylation in old cells, characterized by modifications in cell proportions and metabolism, and activation of time-dependent apoptotic processes. Additionally, Grigoryan et al.’s research [52] demonstrates that aged mouse HSCs exhibit an enlarged nuclear volume and modified nuclear shape. It has been demonstrated that these changes are connected to decreased levels of the nuclear envelope protein Lamin A/C in old HSCs. The authors also noted that the H3K9me2 heterochromatin mark’s distribution in old HSCs had changed. Intriguingly, therapy with CASIN (a Cdc42-activity inhibitor) returned nuclear volume, Lamin A/C levels, and H3K9me2 peripheral localization to the levels seen in young HSCs. Djeghloul et al. [86] have noted changes to the H3K9me3 heterochromatin mark in aged HSCs. One of the key enzymes involved in heterochromatin formation, the methyltransferase SUV39H1, has been demonstrated to be negatively correlated with decreased expression in aged murine and human HSCs. These findings collectively imply the existence of widespread alterations in the nuclear structure of aging HSCs, which may be related to alterations in chromatin structure and gene expression [87,88].

The loss of H4K16ac polarity in aged HSCs linked to increased activity of the small RhoGTPase Cdc42 is another aging-associated epigenetic modification that has just been identified but is still poorly understood. Additionally in this instance, it has been demonstrated that functional HSC rejuvenation correlates with Cdc42 activity suppression with CASIN, which results in the restoration of H4K16ac polarity [50]. In keeping with the phenotypic expansion of HSCs seen with aging, the same group has demonstrated that loss of epigenetic polarity in HSCs is linked to an enhanced rate of symmetric self-renewing divisions in old stem cells [89]. The significance of this observation is highlighted by the part aberrant self-renewal plays in the emergence of AML [90].

A study by Adelman et al. [85] also lends credence to the concept that aging influences leukemogenesis. Analysis of AML-associated epigenetic alterations (differentially methylated areas and enrichment/depletion of various histone marks) and age-associated epigenetic changes in HSC-enriched populations revealed parallels between the two [85]. Moreover, Maegawa et al. [84] highlighted the significance of epigenetic drift in the development of MDS/AML. Progressive hypermethylation of preselected genes has been demonstrated from young to old normal BM, then to MDS, and finally to AML using murine transgenic AML models [66]. It is interesting to note that Mizukawa et al.’s research [91] shows that genetic deletion of CDC42 in murine and human MLL-AF9-induced AML leads to a slower rate of self-renewing divisions and prevents leukemia formation.

The necessity of CDC42 for leukemogenesis suggests that higher activation of CDC42 in aged HSCs may render them more susceptible to leukemic transformation because CDC42 activity is raised following aging in murine and human cells and is associated with poorer HSC function [92]. Additionally, the discrepancies between AML in young and old patients may be explained by CH, which is frequently seen in the elderly. The question of whether clonal hematopoiesis is linked to a worse outcome is, however, still up for debate and has not yet been resolved [93,94,95,96,97].

Last but not least, a number of studies [98,99] have emphasized the crucial function of histone acetyltransferase p300 in promoting malignant transformation and its function as a key driver of the senescent phenotype.

### 3.2. Acute Lymphoblastic Leukemia

The progression of B-ALL is age dependently influenced by the BM populations, particularly macrophages, according to research by Zanetti et al. [100]. Between macrophages from young and old mice, they found clear variations in genome-wide gene expression and chromatin accessibility. These genes and DARs point to a heightened inflammatory response in immature macrophages deriving from BMM. They link the CXCR5-CXCL13 axis to the progression of B-ALL, while direct cell interaction between macrophages and leukemia cells cannot be ruled out as a contributing factor. The CXCR5-CXCL13 axis was suggested as a possible target for treating human B-ALL or as a prognostic marker.

## 4. Aging and Lymphoma

Innate and long-term adaptive immune responses to exogenous antigens and vaccines are primarily coordinated at lymph nodes, which are carefully organized structures of the peripheral lymphoid organs. Additionally, they have a role in immunological tolerance. In addition to changes in the production of chemokines and cytokines required for immune cell proliferation, survival, and function, impaired naive T- and B-cell homeostasis, and a shorter humoral response, aging of lymph nodes causes a decrease in cell transport to and within the nodes, a disruption in the structure and organization of nodal zones, incorrect placement of specific immune cell types, and impaired intercellular interactions. As the lymph nodes get older, their size and quantity decrease, and their structure becomes chaotic [101].

### 4.1. EZH2 and Lymphoproliferative Diseases

The multi-subunit polycomb repressive complex (PRC)-2 is made up of the proteins EED, SUZ12, and RBBP4 in addition to the polycomb group (PcG) protein EZH2. PRC2 initiates polycomb-mediated gene repression by a multitude of methods. Chromatin compaction, which prevents transcription factors from accessing and acting on DNA, and direct block of the transcriptional machinery via inhibition of RNA polymerase II are two of these methods [102]. By serving as a platform for the recruitment of DNA methyltransferases, EZH2 has also been proposed to enable DNA methylation. The histone mark H3K27me3 is crucial because, after being trimethylated by the suppressor of variegation 3–9, enhancer of zeste, and trithorax (SET) domain of EZH2, it serves as a docking site to draw in a second polycomb complex, PRC1, which maintains gene repression by ubiquitinating H2AK119 [103,104]. In a study, authors discovered senescence as a hidden tumor feature defined by a senescence gene signature that was unexpectedly enriched in noncancerous cells through integrative analysis of single cell and bulk transcriptome data from multiple datasets of solid cancer patients [105]. They also discovered two distinct senescence-associated subtypes based on unsupervised clustering. In comparison to patients with the aggressive subtype, those with the senescence subtype exhibited larger tumor mutation burdens and a better prognosis. They developed the senescore scoring system using machine learning, which is based on six signature genes: ADH1B, IL1A, SERPINE1, SPARC, EZH2, and TNFAIP2. In 2290 gastric cancer samples, a higher senescore showed a strong predictive potential for prolonged overall and recurrence-free survival. This finding was independently confirmed by the multiplex staining analysis of gastric cancer samples on the tissue microarray. Surprisingly, the senescore signature proved to be a trustworthy indicator of the effectiveness of both chemotherapy and immunotherapy, with high-senescore patients benefiting from immunotherapy and low-senescore patients responding to chemotherapy [105].

Overexpression of mutant EZH2 has been identified in a variety of B- and T-cell lymphoproliferative diseases in hematological malignancies [106,107,108,109]. A significant study revealed a sizable cohort of EZH2 variant-positive diffuse large B-cell lymphoma (DLBCL) and follicular lymphoma cases. Respectively 7.2% and 21.7% of the cases of follicular lymphoma and DLBCL had “gain-of-function” point mutations that caused a transition from tyrosine to histidine in codon 641 (Tyr641) in the catalytically active SET domain of the EZH2 protein [108]. The germinal center (GC) cell phenotype was present in all of the DLBCL cases and they all looked to be heterozygous for the variation. This study showed that Tyr641 mutations were linked to a significant decrease in EZH2 enzymatic activity in vitro, in apparent contradiction to the elevated EZH2 mRNA levels seen in breast and prostate cancer. According to a theory [108], these mutations may modify the specificity of the EZH2 target gene and thus alter the DNA methylation at PcG sites in these malignancies.

**Figure 2:**
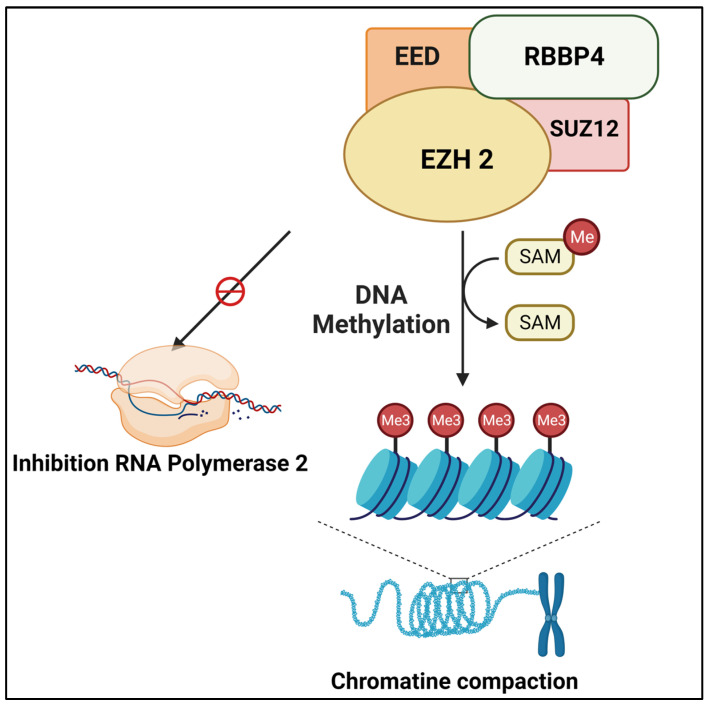
The multi-subunit polycomb repressive complex (PRC)-2 promotes polycomb-mediated gene repression through different mechanisms like chromatin compaction, direct inhibition of the transcriptional machinery via RNA polymerase II, and recruitment of DNA methyltransferases. EED: Embryonic ectoderm development, SUZ12: *SUZ12* Polycomb repressive complex 2 subunit, RBBP4: Histone-binding protein RBBP4, EZH2: Enhancer of zeste homolog 2.

A later investigation, however, demonstrated that the Tyr641 EZH2 mutation conferred an abnormal functional interaction between mutant and WT gene products. Sneeringer et al. demonstrated that the Tyr641-mutant B-cell lymphoma’s malignant behavior results from altered cooperation between the mutated and WT EZH2, which has a general “hyper-trimethylating” effect on H3K27me3 and causes gene repression [110]. This means that the EZH2 Tyr641 mutation may really represent an atypical “gain-of-function” mutation. When compared to naive B-cell controls, GC B cells exhibit a markedly increased level of EZH2 expression and, intriguingly, the transcriptional regulatory profile of EZH2 in GC B cells seems to be closely akin to that of human ESCs [111] (Figure 2).

### 4.2. Hodgkin Lymphoma

In developed nations, Hodgkin lymphoma (HL) is the most prevalent type of malignant lymphoma that affects people under the age of 30 [112,113]. Adolescent and young adult (AYA) HL patients (defined as 15–39 years of age at diagnosis) who are monozygotic (MZ) twins have a risk that is approximately 100 times higher than that of the general population, whereas dizygotic (DZ) twins of patients have a risk that is approximately 7 times higher than that of non-twin siblings [114]. Genetic risk variations in the HLA region, as well as in the genes for IL13, REL, PVT1, GATA 3, and TCF3, have been discovered by genome-wide association studies (GWAS) and collectively explain less than 5% of genetic risk [115,116]. Furthermore, numerous specific DNA modifications have been found in HL tumors [117,118] and cell lines [119], suggesting that epigenetic changes may play a significant role in the development and progression of Hodgkin lymphoma.

Research that used unaffected twins of cancer survivors as genetically and chronologically matched controls evaluated DNAm in long-term AYAHL survivors [120]. Limited to DNA from blood specimens, they found an epigenetic age acceleration in AYAHL survivors relative to their unaffected co-twins in every stratum (sex, age at and since diagnosis, and histological subtype). Survival and unaffected co-twins’ blood DNAm ages varied (64.1 vs. 61.3 years, respectively), mainly in females. No changes in saliva DNAm ages were found. Seventy-four (in blood DNA) and six (in saliva DNA) sites were differently methylated in survivors and co-twins, respectively [120]. These findings imply that AYAHL survivors continue to age epigenetically even after receiving an HL cure. It is probable that therapy-related DNA changes may be a factor in the long-term health effects. It is noteworthy in the study that the AYAHL discordant twin pairs’ different DNA ages were maintained for decades after treatment. According to certain publications, exposure to vinca alkaloids like vincristine, alkylation drugs like dacarbazine, radiation, and bleomycin—all components of the standard AYAHL therapy—may be linked to epigenetic aging [121].

Given that at least three of the four chemicals in conventional chemotherapy and radiation therapy result in epigenetic change, the difference in DNAm age between survivors and their twins is therefore most likely the effect of treatment [122]. Alternative theories include the possibility that the disease is to blame for the accelerated epigenetic age or that the risk factor was acquired before the diagnosis.

### 4.3. Chronic Lymphocytic Leukemia

The accumulation of mature-appearing CD19+ CD23+ CD5+ B cells in the bone marrow, peripheral blood, and lymphoid organs is a hallmark of chronic lymphocytic leukemia (CLL), an indolent B-cell cancer. Similar to the majority of cancers, CLL is a heterogeneous disease with a number of known genetic alterations, including the 17p deletion (del [17p]), the tumor protein 53 (TP53) mutation, and the 11q deletion (del [11q]), all of which have been identified as poor prognostic indicators in patients who have received chemoimmunotherapy [123,124].

Previous studies [125,126,127,128,129] have employed DNA methylation to divide CLL patients into prognostic groupings for overall survival. Additionally, age-related DNA methylation signatures, or biological clocks, have been linked to a risk of mature B cell neoplasms [130,131], indicating that these indicators of biological age may be helpful to forecast cancer risk.

In a publicly available dataset, a study [132] investigated the relationship between epigenetic age acceleration and time to CLL relapse. Before starting chemoimmunotherapy, the Infinium HumanMethylation450 BeadChip was used to analyze the DNA methylation of 35 CLL patients. Blood DNA methylation levels were used to estimate four epigenetic age acceleration metrics: intrinsic epigenetic age acceleration (IEAA), extrinsic epigenetic age acceleration (EEAA), PhenoAge acceleration (PhenoAA), and GrimAge acceleration (GrimAA). To evaluate the relationship between each epigenetic age parameter and time to CLL relapse, receiver operating characteristic curve analysis, and quantile, logistic, and linear regression were used. The likelihood of a CLL relapse was inversely correlated with EEAA and PhenoAA, and favorably correlated with GrimAA. When EEAA and GrimAA were assessed simultaneously in male patients, individuals who relapsed early were differentiated from patients who relapsed later. With IEAA, no relationships were found. These results imply that the time to CLL relapse is correlated with epigenetic age acceleration prior to the start of chemotherapy [132]. These findings offer fresh understanding of the relationship between aging-related DNA methylation alterations and CLL recurrence, and could potentially serve as indicators for treatment relapse and therapy choice.

Other investigations assessed the detection of epigenetic features linked to treatment response, while these studies focused on the risk stratification and prognostic significance of epigenetic markers in CLL.

Although age-related epigenome changes in other diseases have been linked to disease recurrence in the past, offering new insight into how biological aging affects the clinical course of disease [133], similar connections in CLL have not been thoroughly documented. Age-related changes to the epigenome and biological clocks may serve as predictive markers for treatment response and, maybe, may influence therapy choice because the disease typically affects older people. In order to determine the relationship between four biological clocks calculated from blood DNA methylation and time to relapse in CLL patients, a study looked at these variables.

Global DNA methylation profiling of large CLL cohorts and normal B-cell subsets, employing both microarrays and whole-genome bisulfite sequencing, has provided new information [134,135]. Using genome-wide analysis, patients with CLL may be divided into three separate epigenetic subclasses: naive B-cell-like CLL (n-CLL), memory B-cell-like CLL (m-CLL), and intermediate CLL (i-CLL). These subclasses partially represent the stage of B-cell maturation from which the malignancies in the patients’ bodies are produced. Five epigenetic DNA methylation indicators were shown by Queiros et al. to be highly accurate at categorizing patients into these epigenetic groupings [127]. In retrospective studies of mostly early-stage patients, the authors have established the usefulness of this classification approach for estimating time to first therapy and overall survival (OS) [127] (Table 1).

Naive B-cell-like CLL (n-CLL), memory B-cell-like CLL (m-CLL), and intermediate CLL (i-CLL), each with a different time to first therapy and overall survival, can be distinguished in early-stage patients by DNA methylation profiling. However, it is unknown whether DNA methylation can identify people who will respond well to CIT. Using pyrosequencing and microarray data, a study divided treatment-naive patients from three UK chemo and CIT clinical trials into the three epigenetic subgroups, and conducted extensive survival analysis [128]. In IGHV-unmutated (IGHV-U) cases, the n-CLL, i-CLL, and m-CLL signatures were discovered in 80% (n 5 245/305), 17% (53/305), and 2% (7/305), respectively, and in 9%, (19/216), 50% (108/216), and 41% (89/216), respectively. m-CLL was found to be an independent prognostic factor for both progression-free survival and overall survival in CLL patients by multivariate Cox proportional analysis. m-CLL is identified as an independent sign of prolonged survival and may help in the selection of patients likely to display prolonged survival after CIT in the study of epigenetic subgroups in patients enrolled in three first-line UK CLL trials [128].

## 5. Future Perspectives

After allogeneic hematopoietic cell transplantation (allo-HCT), immune escape is a primary cause of AML relapse, with HLA class II expression decrease in leukemia cells accounting for up to 40% of relapses. Relapse has come to be seen as the primary barrier to the full success of allo-HCT for AML in recent years [136,137]. This has sparked studies devoted to comprehending its mechanics [138,139,140]. At the time of diagnosis, defects in this route are rare in AML, but they frequently recur after allo-HCT (1), supporting the hypothesis that recurrence happens when the tumor learns how to avoid T cell-mediated detection. In particular, it has been discovered that, after transplantation, AML blasts change their HLA asset in two ways: either they lose one HLA haplotype genetically, which nearly always includes incompatible HLAs [140], or they stop expressing HLA class II molecules on the surface [138,139]. This second modality in particular appears to be mostly nonoverlapping with genomic haplotype loss in the case of HLA-incompatible transplants and happens with comparable frequency in both cases. For example, only one of the 14 relapses from a study [138] with class II downregulation showed signs of HLA loss, raising the question of which event took place first and was dominant in causing immunological escape. Additionally, some researchers have looked into the epigenetic basis of this immunological escape mechanism and found no indication of fresh mutations in immune-related genes in cases of HLA class II downregulation [138,139].

A study identified polycomb repressive complex 2 (PRC2) as a significant epigenetic driver of this immune escape modality. According to the authors, chromatin accessibility is decreased in a PRC2-dependent manner in conjunction with the loss of HLA class II molecule expression [141]. In vitro and in vivo, pharmacological suppression of PRC2 subunits improves HLA class II expression in AML relapses, leading to a restoration of leukemia recognition by CD4+ T cells. These findings reveal a brand-new connection between leukemia immune escape and epigenetics, which could quickly lead to creative ways to treat or stop AML post-transplantation relapse.

PRC2 is a brand-new and optimistic therapeutic target that has the potential to reverse the resistance mechanism and restore the beneficial graft-versus-leukemia impact. Several drugs that target EZH2 and other PRC2 subunits are now undergoing clinical trials, with GSK126 and EPZ-6438/tazemetostat being the most advanced [142,143]. Notably, mice treated with the substance for 3 weeks did not exhibit obvious side effects, and plasma concentrations of tazemetostat recorded in humans [144,145] without severe toxicities were equivalent to those employed in tests.

Even though in in vitro and in vivo experiments the recovery of HLA class II expression upon PRC2 inhibition did not reach the same levels documented at diagnosis, it is possible to speculate that, in the clinical setting, longer exposure to the compound and synergistic effect with cytokines released by activated T cells may further enhance HLA class II upregulation. In line with this hypothesis, preclinical and clinical studies with PRC2 inhibitors in solid cancers have already evidenced activation of immune responses as one of the mechanisms of action [146,147]. Moreover, better results in terms of HLA class II recovery may be obtained with more potent EZH2 inhibitors or by simultaneously blocking multiple PRC2 subunits, targeting a larger proportion of AML cells and thus avoiding the selection of resistant subclones [148,149].

Finding biomarkers, such as epigenomic markers linked to particular pathologic processes, may help us better understand therapy-resistant cancer and may support hematological precision medicine efforts to choose individualized treatment plans to increase patient survival due to the aging population and the tendency of cancer to primarily affect older people.

FTO alpha-ketoglutarate dependent dioxygenase (FTO), the first discovered m6A demethylase, has been shown to be highly expressed in some subtypes of AML and promotes pro-survival signaling as well as blocking myeloid differentiation by targeting a number of genes, including ASB2, RARA, MYC, and CEBPA, in an m6A-dependent manner. Additionally, FTO maintains aerobic glycolysis in leukemia cells by positively regulating the glycolytic genes PFKP and LDHB [150].

When FTO is aberrantly overexpressed in solid tumors such glioblastoma, breast cancer, and pancreatic cancer, it also acts as an oncogene [151]. Experimental data showing that FTO knockdown effectively slows tumor growth, reduces cancer cell metabolism, and enhances cancer cells’ receptivity to medication therapy strongly suggest that FTO is a prospective therapeutic target for the treatment of cancer in elderly individuals [150]. These findings have led to intensified efforts in the search for potent small-molecule FTO inhibitors. Su et al. discovered two highly effective small-molecule FTO inhibitors, CS1 (also known as Bisantrene) and CS2 (also known as Brequinar), which exhibit powerful anti-tumor effects in both in vitro and in vivo settings in both AML and solid tumors where FTO is highly expressed (such as glioblastoma, breast cancer, and pancreatic cancer) [152]. CS1 and CS2 decrease FTO activity and signaling by obstructing the catalytic pocket and interfering with the binding of FTO to m6A modified targets. Importantly, even at doses four times greater than those used to treat cancer, administration of CS1 or CS2 to C57BL/6 mice resulted in negligible drug toxicity.

For elderly AML patients who cannot tolerate intense chemotherapy, hypomethylating drugs (HMAs) are frequently utilized as frontline therapy. However, due to the elevation of immune checkpoint gene expression and associated immune evasion, the majority of HMA-treated patients eventually acquire drug resistance. By inhibiting the expression of the immunological checkpoint gene LILRB4, CS1 and CS2 therapies make AML cells more susceptible to T cell cytotoxicity. This finding supports FTO inhibition as a useful tactic to combat immune evasion brought on by HMAs. The ability of leukemia stem/initiating cells (LSCs/LICs), the main population thought to be responsible for treatment failure and disease relapse in AML, to self-renew is likewise noticeably reduced by pharmacological inhibition of FTO with the two drugs or genetic depletion of FTO.

There is a growing belief that the epigenetic composition dramatically changes as an organism ages. Various cell types and tissues can show age-related alterations in DNA methylation [153]. The so-called “epigenetic clock” may accurately predict the donor age because of the great repeatability of age-associated DNA methylation changes [154]. Notably, studies linking the pace of epigenetic aging to life expectancy have shown that age-related DNA methylation can also be a reflection of biological aging [155]. It is particularly remarkable that reprogramming into induced pluripotent stem cells completely resets age-associated DNA methylation patterns [156]. However, age forecasts do not always work when it comes to cancer tissue.

The epigenetic clocks appear to be accelerated in the majority of malignancies but decelerated in others [157]. This may be explained by the fact that healthy tissue’s ability to predict age is based on a cross-section of many cells from the regularly developing organism, whereas tumor tissue recapitulates the epigenetic composition of the tumor-initiating cell. In fact, there is proof that patient-specific, age-associated DNA methylation patterns can be utilized to monitor clonal proliferation [158].

Therapies that inhibit EZH2 may accelerate the exhaustion of CSC populations since data suggest that EZH2 plays a function in promoting self-renewal in at least some cancers (including some hematological malignancies) [159]. Furthermore, it would be predicted that decreasing EZH2 might have therapeutic benefits if it had non-stem cell specific carcinogenic effects, such as increasing cell proliferation or repressing differentiation. As a result, much effort has been put into developing EZH2 inhibitors. The first medication to be suggested to inhibit EZH2 is 3-deazaneplanocin (DZNep), which exerts its indirect action by competitively inhibiting S-adenosylhomocysteine hydrolase. Adenosylhomocysteine, an enzyme substrate, builds up as a result, which inhibits methyltransferases, causes the PRC2 complex to degrade, and lowers EZH2 levels [160,161].

A model system and novel target for the development of EZH2 inhibitors has been made available by the identification of EZH2 mutations in lymphoma, particularly DLBCL [162,163]. In their study explaining how microRNAs control EZH2, Zhao et al. combined DZNep first with the inhibitor of histone deacetylation Vorinostat and then with the inhibitor of the bromodomain and extra-terminal (BET) domain JQ1 [164]. By disrupting the c-MYC-miR-EZH2-HDAC3 feedback loop, this medication combination boosted the expression of the tumor suppressor miR-29, downregulated its target genes, and inhibited the formation of lymphomas. An Ezh2-selective small-molecule inhibitor called EI1 was created by Qi et al. [165] that competitively binds to the S-adenosylmethionine (SAM) pocket of the Ezh2 SET domain in both Tyr641-mutated cells and WT cells. The Ezh2 mutant cells underwent death, G1 growth arrest, and differentiation into memory B cells as a result of this suppression of histone H3K27me3. It is not a well-targeted therapy because there is evidence that some of these drugs have a broad inhibitory effect on protein methyltransferases other than EZH2 [160].

The finding that the introduction of transcription factors associated with embryonic stem (ES) cells into terminally differentiated somatic cells can transform their functionality into an ES cell state, a process accompanied by the erasure of the epigenetic parameters of the mature somatic cell, has stoked interest in the transcriptional and epigenetic characteristics that define cell identity [166,167].

## 6. Conclusions

Recent research has proven the potential for more direct cellular reprogramming, from one somatic cell type to another [168]. Reprogramming technologies have a lot of potential from a hematological standpoint because it might be challenging to obtain eligible donors for bone marrow transplantation and, occasionally, enough HSCs. Somatic cell reprogramming has thus been extensively researched as a method of producing transplantable HSCs, either directly or via developing induced pluripotent (iPS) cells into HSCs. Despite the fact that a few studies [169,170,171] were able to develop transplantable HSCs with long-term activity from fibroblasts and iPS cells, the majority of experiments were unable to do so. Given that HSCs cultivated in vitro quickly lose their stemness, the reliance on in vitro culture techniques in such procedures may help to explain this. This issue was recently highlighted by Riddell et al. [172], who discovered that terminally differentiated blood cells might be converted into transplantable, functional HSCs by being briefly exposed to a number of HSC-specific transcription factors in vivo.

However, it seems conceivable that changed expression of a few important aging loci could cause HSC aging. If this is the case, normalizing these age-dysregulated loci should offer a way to revive aging HSCs in terms of function. This theory was recently tested in a study that used blastocyst complementation to redifferentiate old hematopoietic stem and progenitor cells (HSPCs) into HSCs in vivo [47].

When examined for a number of known age-related functional flaws, the function of the resultant HSCs was found to be strikingly similar to that of young HSCs and failed to resemble aged HSCs [47]. Because of this, the HSC aging state seems to be reversible and predominantly relies on a changed transcriptome and epigenome.

However, scientific findings support proof-of-concept studies by demonstrating that it is possible to modify the HSC aging state by using exogenous agents. Additionally, researchers might be able to use HSC aging-preventive therapy in addition to intervening with an already established aged disease and eventually acquire a more complete grasp of the correct targets.

To do this, it is crucial to understand the difference between aging that affects only one organ and aging that affects an entire person. Due to the fact that age progression is a complex process, its blockage or even reversal will probably necessitate the risky manipulation of one or more important tissue homeostasis regulators. For instance, the tumor suppressor Trp53 has a well-established role in increasing aging [173] but interfering with such a potent tumor suppressor would almost certainly result in the growth of a cancer [174].

In conclusion, hematopoietic stem cell phenotypes associated with aging, such as leukemia and lymphoma, are influenced by the aging of the hematopoietic system and the stem cell niche. Because epigenetic mechanisms are reversible, understanding these changes will open up interesting avenues for therapeutics against age-related illnesses. Furthermore, the advancement of high-throughput epigenome mapping technologies will enable the identification of the “epigenomic identity card” of each and every patient as well as hematological disease, opening the door to the discovery of novel molecular biomarkers for diagnosis, prediction, and prognosis.

## Figures and Tables

**Table 1:** Completed clinical trials about hematological malignancies in relation to aging.

Study Title	Conditions	Study Type	Sex	Age	NCT Number
Characterization of Proliferating Compartment in B-Cell Patients and in Healthy Aging Subjects	Chronic Lymphocytic Leukemia	Observational	All	18 Years and Older (Adult, Older Adult)	NCT01110863
Exercise Training to Promote Resilience to Chronic Lymphocytic Leukemia	Aging; Chronic Lymphocytic Leukemia	Interventional	All	18 Years and Older (Adult, Older Adult)	NCT04950452
Bryostatin 1 and Rituximab in Treating Patients With B-Cell Non-Hodgkin’s Lymphoma or Chronic Lymphocytic Leukemia	Leukemia; Lymphoma	Interventional	All	18 Years and Older (Adult, Older Adult)	NCT00087425
A Patient-centered Communication Tool (UR-GOAL) for Older Patients With Acute Myeloid Leukemia, Their Caregivers, and Their Oncologists	Acute Myeloid Leukemia	Interventional	All	60 Years and Older (Adult, Older Adult)	NCT04625413
Bryostatin 1 and Interleukin-2 in Treating Patients With Refractory Solid Tumors or Lymphoma	Lymphoma; Small Intestine Cancer; Unspecified Adult Solid Tumor, Protocol Specific	Interventional	All	18 Years and Older (Adult, Older Adult)	NCT00003993
Lymphoma Follow-up	Lymphoma	Observational	All	21 Years and Older (Adult, Older Adult)	NCT00744120
Bendamustine + Rituximab in Older Patients With Previously Untreated Diffuse Large B-cell Lymphoma	Diffuse Large B-Cell Lymphoma; Diffuse Large-Cell Lymphoma	Interventional	All		NCT01234467
The Effects of Anthracycline-based Chemotherapy on Peripheral Vascular Function	Breast Cancer; Lymphoma; Chemotherapy Effect	Observational	All		NCT03062878

## Data Availability

Not applicable.
